# The Royal Netherlands Football Association (KNVB) relative age solutions project—part two: an adapted e-Delphi study

**DOI:** 10.3389/fspor.2025.1565819

**Published:** 2025-05-29

**Authors:** Adam Leigh Kelly, Frederike Zwenk, David Mann, Jan Verbeek

**Affiliations:** ^1^ Research for Athlete and Youth Sport Development (RAYSD) Lab, Birmingham City University, Birmingham, United Kingdom; ^2^ The Royal Netherlands Football Association (KNVB), Zeist, Netherlands; ^3^ Department of Human Movement Sciences, Vrije Universiteit Amsterdam, Amsterdam, Netherlands; ^4^ Department of Psychology, Faculty of Behavioural and Social Sciences, University of Groningen, Groningen, Netherlands

**Keywords:** relative age effects, talent identification, talent development, athlete development, youth soccer, youth football

## Abstract

**Introduction:**

Following the lack of widely implemented interventions to mitigate Relative Age Effects (RAEs) in sports, the Royal Netherlands Football Association (KNVB) called on stakeholders to propose relative age solutions in youth soccer (Part One). This initial study yielded 13 lower-order potential solutions, many of which remain hypothetical. Therefore, this study aimed to evaluate these solutions to overcome RAEs in youth soccer using a two-round adapted e-Delphi study.

**Methods:**

Fifteen international experts, including both researchers and practitioners, rated (out of 9) each solution on how likely it is to directly and indirectly mitigate RAEs (Round 1) and how feasible it is to implement (Round 2).

**Results:**

Findings indicated that “rotating cut-off dates” was perceived as the most effective solution to mitigate direct and indirect RAEs (6.2 ± 1.6), although it was not rated particularly feasible (4.6 ± 2.5). In comparison, while “cueing differences in age” was perceived as the most feasible solution (6.7 ± 2.1), it was deemed less useful for mitigating RAEs (5.2 ± 2.3). Taken together, “cueing differences in age” was considered the most viable solution across both rounds (5.8 ± 2.3).

**Discussion:**

Interestingly, highly rated solutions perceived to effectively moderate RAEs were generally expected to be more challenging to implement. Results also showed regular disagreement amongst the international experts, highlighting that creating consensus on possible relative age solutions may be difficult to achieve in youth soccer. Moving forward, the highest rated solutions should be designed, implemented, and evaluated based on their effectiveness and feasibility in practice.

## Introduction

Over the last four decades, there has been widespread research attention on Relative Age Effects (RAEs) in sport. Following the seminal works of Grondin et al. (1) and Barnsley et al. (2), several reviews [e.g., (3–5)], books [e.g., (6, 7)], and a substantial amount of empirical studies in different sports have been published since; ranging from chess [e.g., (8, 9)] to basketball [e.g., (10, 11)] and cricket [e.g., (12, 13)] to synchronized swimming (14). Youth soccer has emerged as a regularly researched area where RAEs are highly prominent and persistent [e.g., (15–18)]. Such studies consistently show that age differences resulting from cut-off date eligibility (e.g., U12, U13, U14, etc.) favour relatively older players (i.e., those born near the start of the cut-off date), while simultaneously disadvantaging relatively younger players (i.e., those born near the end of the cut-off date). During this time, however, there is a lack of proposed solutions that have been designed, implemented, and evaluated to test their effectiveness and feasibility to mitigate RAEs (19). Given its worldwide popularity coupled with the early selection procedures often applied in high performance environments (e.g., academies), soccer provides an important context to evaluate the effectiveness and feasibility of relative age solutions.

Research in sport has emphasised multiple potential implications of RAEs, including higher dropout rates and exclusion of relatively younger athletes at higher competitive levels. These inferences are manifested in the skewed birth date distributions of athlete populations and are likely limiting the pool of potential talent. Any attempt to implement solutions into practice to mitigate RAEs requires a thorough understanding of the fundamental mechanisms of what exactly causes them (20). To date, research has put forward several theoretical frameworks that aim to explain the emergence of RAEs in sport [e.g., (3, 12, 21, 22); see Part One for an overview, (23)]. While these frameworks offer different views, the general consensus is that RAEs arise from an interaction between various factors, such as superior physiological capacities, an older training age, and advanced cognitive development. This coincides with the evaluation of these advantages by social agents (i.e., coaches, parents, and peers), whereby coaches (and other scouts/recruiters) who act as gatekeepers to future developmental opportunities often misinterpret physiological advantages, prolonged training, and/or enhanced cognitive abilities as talent (3, 21). This bias seems to underpin RAEs by creating a self-fulfilling prophecy, as those initially considered as more talented (i.e., relatively older players) are provided access to better coaches and more resources, further increasing their opportunities for future selections.

Given the theoretical mechanisms, it becomes apparent why RAEs are especially prevalent across youth levels in sport, and particularly soccer. In soccer, it is common to group players based on chronological age (4). As a result, 12- or 24-month age differences might occur between the youngest and oldest player in an age-grouped team. Especially across the youngest age groups (e.g., U8, U9, U10, etc.), such an age difference represents a substantial part of a player's developmental career. For instance, up to the age of 10 years, a 12-month age gap represents more than 10% of the player's total lifespan. When combined with selection and grouping procedures involving the evaluation or judgement of performance and potential, which generally already begins during childhood (e.g., aged 8, 9, 10, years) (24), these age differences result in a substantial overrepresentation of relatively older players at representative levels such as academy teams (25).

In an attempt to simplify how RAEs arise, Mann (26) classified two general working mechanisms as “direct” effects (i.e., benefits experienced by relatively older players themselves) and “indirect” effects (i.e., benefits experienced by relatively older players through others). Although this distinction supports the practical implementation of solutions, there remains a lack of research that has tested solutions to mitigate RAEs in practice. Webdale et al. (19), for example, provided a valuable synopsis of the possible benefits and drawbacks for proposed relative age solutions in sport. Importantly, though, the utility of many of these solutions remains largely generalised and mainly hypothetical, as very few have been implemented or empirically studied across different sports. Indeed, it is important to recognise different sports require different approaches to RAEs. For instance, grouping based on chronological and biological age may be more suitable for team sports (e.g., basketball, rugby, soccer), whereas birthday-banding, corrective adjustments, and proficiency level-based competition may be more useful for racket (e.g., badminton, squash, tennis), timed (e.g., cycling, sprinting, swimming), and combat (e.g., boxing, judo, taekwondo) sports, respectively (27). Further research is required, however, to better understand sport-specific relative age solutions and substantiate these examples.

In an attempt fill the relative age solutions void and fulfill the need for sport-specific measures, the Royal Netherlands Football Association (KNVB) created a project to better understand potential approaches to mitigate RAEs in soccer, with the long-term goal of designing, implementing, and evaluating viable interventions in Dutch youth soccer settings. As a first step in this project, stakeholders were invited to propose relative age solutions in youth soccer, which resulted in 13 lower-order solutions from three higher-order themes: (a) altering the behavior of observers (*n* = 3), (b) implementing rules when selecting teams (*n* = 6), (c) adjusting competition structures (*n* = 4) [see Part One (23);]. Interestingly, no new suggestions outside the existing literature were proposed in any of the participants' submissions. Whilst no new proposals were suggested, to our knowledge, only two have been empirically tested in soccer to date [i.e., “cueing differences in age”, (28); “grouping based on chronological and biological age”, (29)]. Out of the 143 proposed solutions, results showed the most frequent higher-order theme that was put forward by the participants was “adjusting competition structures” (*n* = 78), with “modifying age bands” (*n* = 25) the lower-order solution that was suggested most often.

The second step of this project, and the corresponding aim of this present study, was to evaluate the direct and indirect “effectiveness” (i.e., the likelihood that a solution would be successful in mitigating the direct and indirect RAEs in soccer) and the “feasibility” (i.e., the practicality and possibility to design and implement a solution in youth soccer) of these 13 potential solutions. Using “direct” and “indirect” as distinctions for effectiveness provided a useful framework to classify solutions that might be effective based on the underlying mechanisms of RAEs they address, serving as a useful input as part of a modified electronic Delphi (e-Delphi) approach. The e-Delphi technique is commonly used to increase the understanding of complex phenomena without much conclusive information (30). As it became apparent in Part One of the current project, many solutions have been suggested to mitigate RAEs. To date, though, there is a lack of a systematic evaluation of their utility and currently limited to the context of a single sport or country [e.g., (28, 29)]. Typically, evaluating such interventions would involve scientific techniques such as systematic reviews and meta-analyses; however, the relative lack of empirical investigations on the utility of RAEs solutions limits this approach. As such, given the lack of empirical evaluations across multiple contexts, we deemed the e-Delphi approach as a particularly useful method to evaluate the utility of potential interventions that could mitigate RAEs in youth soccer. The Delphi technique, and the e-Delphi in particular, enables to gather the judgments across a wide pool of international experts, given that all communication is online. Indeed, gathering a mixed pool of experts from different backgrounds enables the simultaneous evaluation of the conceptual mechanisms and practical implications of each solution beyond the national youth soccer contexts in which it has been examined so far.

## Methods

### Participants

Given the global importance of RAEs and our objective to evaluate both the effectiveness and feasibility of proposed solutions, we aimed to create an international expert panel that reflected these areas of expertise. As such, panel members of the e-Delphi were selected through purposeful sampling, considering several aspects to construct the panel (31). With regards to the size, heterogeneity, and expertise level of the panel, we invited 25 researchers who were (co-)authors (excluding the current research team) from book chapters in two published books on RAEs [e.g., (6, 7)], as well as 15 practitioners working within youth soccer who had publicly discussed the impact of RAEs in practice, to participate. This size of the expert panel was considered adequate for the specific topic of RAEs in sport (32). In addition, the heterogeneous sample of both researchers and practitioners reduced the risk of response bias, while also offering a broader picture of the utility of potential RAEs solutions. Based on these criteria, it was anticipated that these participants would have a certain level of interest in the topic, which would motivate them to participate in the e-Delphi study.

After invitations were administered, 15 participants took part in the e-Delphi survey. First-round data from three participants who did not complete the second-round survey were retained to help capture the wider expertise. All participants were informed of the study procedures and provided electronic consent prior to participation. This study received ethical approval from the Health, Education, and Life Sciences Faculty Academic Ethics Committee at Birmingham City University, United Kingdom (ethics code #9524).

### Measures and procedures

The Delphi technique is a systematic method, consisting of a series of surveys, to develop consensus amongst a designated panel of domain-specific experts. While there are no clear guidelines for the design of a Delphi study, typical elements include anonymity amongst the panel members, several iterative rounds, and the analysis of group results (33, 34). In the present study, we used a modified e-Delphi approach, consisting of an electronic approach with *a priori* defined maximum of two rounds. The e-Delphi is particularly suited for expert panels that include international participants from multi-stakeholder groups, enabling them to complete this online at their own convenience.

For the initial round of our e-Delphi, we created an online survey in Microsoft Forms (Microsoft Corp., Redmond, WA, USA) and invited all participants to take part in the study. Here, they were required to rate the direct and indirect “effectiveness” of each of the 13 solutions to mitigate RAEs in youth soccer, derived from the first part of this research project (see the list of solutions in Table 1). In the invitation, participants were asked to provide consent to participate in the e-Delphi and were made aware that their responses would be processed anonymously. Each solution was evaluated using 10 items, resulting in 130 items within the first round. Each item was phrased as a presumption in the following way: “This solution … [mitigates a specific (in)direct effect]”. Thereby, the items were divided into their effectiveness to mitigate “direct” (6 items) and “indirect” (4 items) effects of RAEs. This distinction involved addressing different (dis)advantages considered to arise from RAEs. For instance, “This solution decreases the likelihood of relatively older players experiencing greater levels of self-efficacy” was based on the hypothesised “Galatea Effect”, whereby unjustly raised self-efficacy of relative older players can subsequently enhance performance (21). This distinction enabled the examination of how certain solutions might act on the different mechanisms underlying the emergence of RAEs, whereby the items related to the direct effects are (dis)advantaged experienced through enhanced maturation, whereas indirect effects are disadvantages experienced through the behaviour of social agents. Additionally, it enabled the possibility for hybrid approaches to appear (e.g., complementary approaches where one solution targets direct effects, the other mitigates indirect effects), as well as aggregates the overall mean effectiveness score. Instead of discussing solutions as mutually exclusive, potential solutions could now be considered as complementary because they might differ in effectiveness on either direct or indirect RAEs.

**Table 1:** Descriptive statistics for each solution over the two e-Delphi rounds. These are listed from highest to lowest based on the overall rating.

Solution	Direct Effectiveness			Indirect Effectiveness			Combined Effectiveness			Feasibility			Overall Rating^c^			
	M ± SD	Mdn	KAlph^a^	M ± SD	Mdn	KAlph	M ± SD	Mdn	KAlph	M ± SD	Mdn	KAlph	M ± SD	Mdn	KAlph	Con^b^ (*n*)
Cueing Differences in Age	4.7 ± 2.2	5	−0.01	5.9 ± 2.3	7	0.30	5.2 ± 2.3	5	0.19	6.7 ± 2.1	7	0.15	5.8 ± 2.3	6.5	0.29	5
Grouping Based on Chronological and Biological Age	6 ± 2.2	6	−0.04	5.8 ± 2.1	6	−0.04	5.9 ± 2.2	6	−0.04	5.2 ± 2.5	5.5	0.27	5.6 ± 2.3	6	0.12	
Submitting Entry Exemption	5.5 ± 2	6	0.04	5.3 ± 1.6	6	−0.01	5.4 ± 1.8	6	0.03	5.6 ± 2.3	6	0.19	5.5 ± 2.1	6	0.12	
Rotating Cut-off Dates	6.3 ± 1.6	6	−0.03	6.2 ± 1.6	6,5	−0.03	6.2 ± 1.6	6	−0.03	4.6 ± 2.5	4.5	0.15	5.5 ± 2.2	6	0.17	
Capping the Average Team Age	5.6 ± 2.1	6	0.01	5.7 ± 2.1	6	0.05	5.7 ± 2.1	6	0.02	4.8 ± 2.1	5	0.19	5.3 ± 2.1	6	0.13	
Categorising on Characteristics Other than Age	6.3 ± 2	7	−0.04	5.9 ± 2.1	6	−0.03	6.1 ± 2.1	7	−0.02	4.4 ± 2.3	4	0.19	5.3 ± 2.3	6	0.21	1
Modifying Age Bands	6.1 ± 2	6	−0.04	5.4 ± 2.1	6	−0.06	5.8 ± 2	6	−0.01	4.8 ± 2.6	5	0.08	5.3 ± 2.4	6	0.07	
Applying Player Selection Quotas	4.7 ± 2.3	5	0.00	5.2 ± 2.3	6	0.12	4.9 ± 2.3	5	0.05	5.6 ± 2.5	6	0.09	5.2 ± 2.4	6	0.10	
Delaying Selection and Deselection	5.1 ± 2.3	5,5	−0.03	5.8 ± 2.2	6	0.17	5.4 ± 2.3	6	0.08	4.9 ± 2.7	5	0.12	5.2 ± 2.5	5	0.11	2
Raising Awareness of Relative Age Effects	4.4 ± 2.2	5	0.00	4.7 ± 2.3	5	0.07	4.5 ± 2.2	5	0.03	6.1 ± 2.5	7	0.08	5.2 ± 2.5	6	0.17	
Testing Objective Skills	5.9 ± 1.8	6	−0.02	5.7 ± 1.7	6	0.01	5.8 ± 1.7	6	0.00	4.1 ± 2.4	4	0.31	5 ± 2.3	5	0.29	3
Using Corrective Adjustments	5.1 ± 2.2	5	−0.07	5.8 ± 2.2	6	0.05	5.4 ± 2.2	5	0.01	4.5 ± 2.7	4	0.17	5 ± 2.5	5	0.12	
Shifting Cut-off Dates	3.5 ± 1.8	3	−0.06	3.6 ± 2	4	−0.05	3.6 ± 1.8	3	−0.06	5.3 ± 3	5	0.39	4.3 ± 2.6	4	0.29	1

^a^ Krippendorf's Alpha.

^b^ the items that reached consensus (≥80%).

^c^ the overall rating is the average of all items together, M = Mean, SD = Standard Deviation, Mdn = Median.

Following the first e-Delphi round, participants could immediately commence the second e-Delphi round. While a typical feature of the Delphi methodology is the provision of feedback amongst panelists, we adapted this to reduce participant burden and to lower attrition rate (35). If participants did not begin the second Delphi round two weeks after completion of the first, they received an email invitation that included the results of the first e-Delphi round and a link to commence the second e-Delphi round. This feedback included a bar chart describing the distribution of (anonymous) responses from the first round. The second (final) e-Delphi round aimed to evaluate the “feasibility” of each of the 13 solutions. In line with the first e-Delphi round, ten items were formulated as a presumption reflecting the practical feasibility of that solution to be implemented in the context of youth soccer. Each item was phrased as a presumption in the following way: “This solution … [mitigates a specific (in)direct effect]”. Items included question such as “This solution requires significant financial resources (e.g., extra teams) at an individual club level” and “This solution is expected to yield positive results on a short timescale (i.e., within one season)”.

For both rounds, participants were asked to evaluate the likelihood that the presumption for that specific solution is true, ranging on a Likert scale from 1–9 (1: Not Very Likely; 9: Very Likely). The use of a nine-point rating scale aligns with the established consensus-criteria used in Delphi research and is recommended in the process before establishing definitive consensus, aligning with our study's aim (36, 37). If participants could not reliably evaluate the likelihood of that solution mitigating RAEs, they were instructed to give a score of 0, which were subsequently treated as missing values. In addition, if participants were not fully familiar with the proposed solutions hypothetical working mechanisms, they could revert to a brief one-page summary of each solution that was provided by the research team. Lastly, participants were encouraged to provide qualitative justification for their rating via an optional open textbox provided at the end of each assessment, enabling panellists to comment on the solution as a whole. See Supplementary File S1 for the complete survey.

### Data analysis

Following the conclusion the second e-Delphi round, using the R base package [version 4.4.0; (38)], mean (M) ratings with standard deviations (SD) as well as median (Mdn) and interquartile ranges (IQR) were compiled on items related to their perceived: (a) direct effectiveness to mitigate RAEs, (b) indirect effectiveness to mitigate RAEs, (c) combined effectiveness to mitigate RAEs, (d) feasibility to be implemented in youth soccer, and (e) overall rating, of which was used to rank order the solutions. To examine the consensus amongst the e-Delphi panel, we calculated the percentage agreement among the panellists for each solution. Consensus was defined as 80% of the panellists rating the items between either 1 and 3 (the statement is not true for this solution) or 7 and 9 (the statement is true for the solution). In addition, we also computed Krippendorf's Alpha with the “irr” package in R (39) to assess the inter-rater agreement on each of the items related to RAEs solutions (40). These statistics provided an overview of the variability in expert opinions, and, as such, also served as an indicator of consensus.

Qualitative responses that the panellists provided were also collated, which were analysed by one member of the research team (last author) and then reviewed by another (first author). This included coding the qualitative comments as “nuance” (i.e., the panellist provided opposing arguments to make their quantitative response more nuanced) or “justification” (i.e., the panellist provided supporting arguments to strengthen their quantitative response). The comments were subsequently analysed based on the specific content of their argumentation (e.g., significant need for extra resources, limited applicability in soccer), summarising comments that referred to the same arguments. These data were used in the final analysis to provide insight on the (lack of) consensus for specific solutions, and are included as examples in the results to provide support for the mean group ratings (41). Comments that did not include any information from the panellist on the potential benefits and drawbacks of each solution were not considered further in the analysis.

## Results

**Figure 1:**
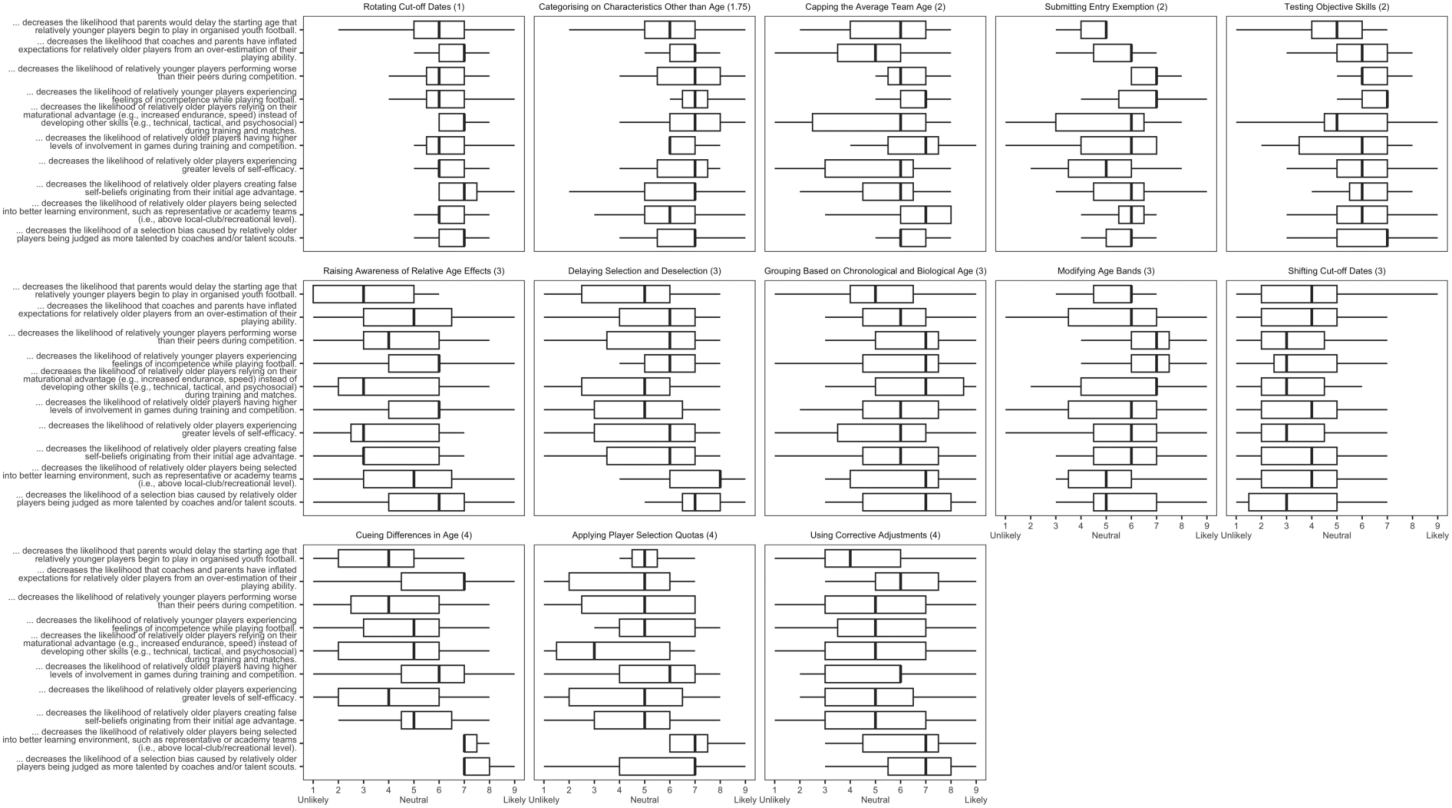
The interquartile ranges for each of the statements for round one (effectiveness), with the subplots ordered according to the overall interquartile range for each proposed solution.

**Figure 2:**
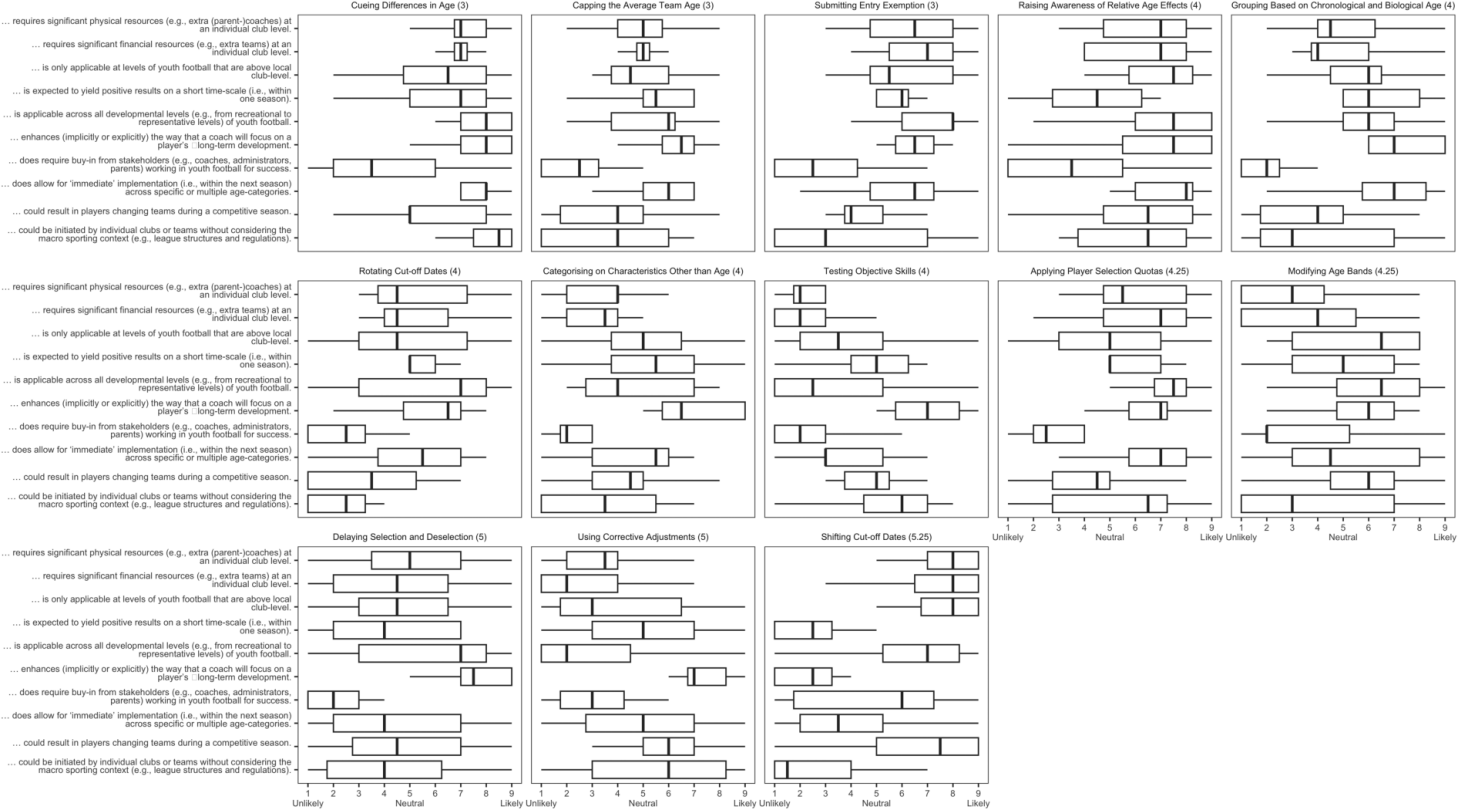
The interquartile ranges for each of the statements for round two (feasibility), with the subplots ordered according to the overall interquartile range for each proposed solution.

Descriptive statistics (i.e., mean, standard deviation, and median) from the panelists during the first (effectiveness) and second (feasibility) e-Delphi rounds are presented in Table 1. Table 1 also presents Krippendorfs alpha as an indicator of the consensus amongst panel members for each of the e-Delphi statements related to each proposed solution. The interquartile ranges for each of the statements are shown in Figure 1 (round one) and Figure 2 (round two). In both figures, the subplots are ordered according to the overall interquartile range for each proposed solution. The perceived benefits and drawbacks for each of the 13 proposed solutions are presented in Table 2. Since it is beyond the scope of this study to provide an in-depth overview from each of the 13 proposed solutions, the qualitative results in this section are presented below for “cueing differences in age”, “rotating cut-off dates”, “categorising on characteristics other than age”, “grouping based on chronological and biological age”, and “submitting entry exemption”. This is because these solutions were considered the most effective to mitigate direct and indirect RAEs as well as the most feasible to be implemented in youth soccer.

**Table 2:** The perceived benefits and drawbacks for each of the 13 proposed solutions. These are listed based on the three higher-order approaches to correspond with Part One of this project [see Figure 1 in (23)].

Approaches	Solutions	Perceived Benefits	Perceived Drawbacks
Changing the Behaviour of Observers	Raising Awareness of Relative Age Effects	I think it is good as an additional concept. The underdog-concept also shows that children can also develop more skills by competition with relative older children. So, I think that a combination of both is good (Participant 10).	Maybe causes another issue where players are being selected on the basis of age rather than actually technical and tactical ability and potential, which really should be the focus. While it would address the RAE issue, I am not sure that it is the best approach to ensuring players are selecting on the factors that are most important (Participant 1).
	Cueing Differences in Age	This is a way to implement something quickly and effectively to raise the age differences that will occur. It may not prevent certain players being disadvantaged during the game, but it will raise awareness of age and possible maturity differences (Participant 2).	This solution looks very promising, although I would love to see some replication of these findings in different labs and contexts to establish the robustness of these effects (Participant 3).
	Testing Objective Skills	I think this is a good call. Having standards where players can be judged not just for their age groups but for their relative age would be good. Equivalent systems, but for variance in maturation and fitness tests, are already in place with the English Premier League academy system allowing coaches to better evaluate strength and weakness of players from a developmental perspective (Participant 1).	For physical attributes, a clear link with relative age has been found. However, for other important attributes like cognitive maturation, the link is far from clear. As a result, skill-testing can only be done for a limited number of attributes (Participant 6).
Implementing Rules when Selecting Teams	Submitting Entry Exemption	The flexibility of our pathways is extremely important due to the non-linear nature of development so yes, I would favour this. There would need to be a lot of player and parental supports available while navigating “playing down”—counteracting perceptions of failure/lack of ability (Participant 6).	A key question here is what criteria are used to allocate a youth player to a higher or a lower team: is it just by month of birth or is also the developmental age considered (Participant 5)?
	Applying Player Selection Quotas	Raising awareness by coaching and scouting staff is important, but many studies show that it is not enough to decrease RAE- and maturity-related bias in soccer. I think it is good to involve in the further education program from coaching and scouting staff. But more solutions are necessary (Participant 10).	The value of this solution lies in the assumption that coaches don't already know about this effect. In my experience, most are aware of it and so education, while feasible and relatively inexpensive, may not be that helpful (Participant 3).
	Delaying Selection and Deselection	I like the idea of delaying the selection/deselection aspect in favour of a focus upon development and managing the journey through puberty more effectively (Participant 2).	Delaying selection is a good idea, but the problem is that the RAE is present from 6 years and holds pretty steady through to mid adolescence (i.e., 15 to 16 years). Even if you select at this point you are still selecting from a biased sample. The impact of RAE upon developmental differences will be arguably less at this time, but the academies are still full of BQ1s (Participant 1).
	Capping the Average Team Age	This option could begin to level out the playing field but may prove difficult across all levels of the game. The idea behind it is a good one (Participant 4).	Bio-banding has absolutely nothing to do with the RAE so while it may be effective at addressing maturational differences it will have no impact whatsoever. Part of the problem with addressing the RAE has been the fact that people are quick to attribute it to differences in maturation when this is clearly not the case (Participant 1).
	Grouping Based on Chronological and Biological Age	This reallocation concepts would be a very good solution for RAE- and maturity-related bias in soccer. Comparing with the concept of bio-banding, we see that the age-differences in different age categories are becoming smaller and better, more realistic (Participant 10).	I don't see throwing in maturation as a viable approach to address the RAE as you are talking about two completely different issues here. Plus, RAE effects are present from early childhood and maturation related biases and advantages do not kick in until approximately 11 years of age (Participant 1).
	Using Corrective Adjustments	As an additional tool in player evaluations then yes, all data should be examined. But how to incorporate it into a score for team selection or to remove performance (dis)advantages is complex (Participant 6).	As mentioned before, it is not clear what this method will look like for team sports. For individual sports, it is clear that there are sprinting times for example that are very representative of the overall performance. In team sports, however, the performance is multi-dimensional, which makes it very hard to understand how this kind of adjustment would be applied (Participant 1).
Adjusting Competitive Structures	Modifying Age Bands	The smaller the age range, the smaller the RAE (Participant 7).	If you just reduce the age band or extend it, then the RAE will still remain [..] it simply shifted from BQ1 and BQ2 to BQ1 and BQ3 (Participant 1).
	Rotating Cut-off Dates	Each player experiences being the youngest and oldest on the team. The overall average of these experiences should minimize, if not eliminate, the RAE. Overall, a very effective proposal (Participant 7).	Cut-off date modification and birthday-banding will not be enough to encounter the RAE bias, as showed in different studies. It's also difficult to implement it organizational in daily life, different systems use different cut-off dates (Participant 10).
	Shifting Cut-off Dates	This is an interesting idea. Players who are disadvantaged in both education *and* sport may benefit from this approach and as such it deserves further investigation, particularly if it supports the social and emotional development of players due to more appropriate peer grouping (Participant 2).	Varying cutoff dates for the same sport in different leagues would be effective, however, varying cutoff dates for different sports will still maintain RAE (Participant 7).
	Categorising on Characteristics Other than Age	These initiatives definitely have merit, although I fear that they'd have to delivered in conjunction with coach education, as changing the mindset for some (i.e., winning at all costs at youth level) will be necessary for successful adoption of such worthy initiative (Participant 6).	Its success will be largely dependent on the clubs having a balanced distribution of players coming into the academy in the first place (Participant 1).

The highest overall ranked solution was “cueing differences in age”. Participants reached consensus on 5 items, and strongly agreed that this solution decreases the likelihood of a selection bias caused by relatively older players being judged as more talented by coaches and/or talent scouts (7.2 ± 1.8; IQR = 1; % _agree_ = .87). Related to this, consensus was reached amongst participants that this solution would to some extent address “the likelihood of relatively older players being selected” (6.7 ± 1.7; IQR = .5; % _agree_ = .80). For example, Participant 4 stated: “Additional information (such as shirt numbering) will provide greater clarity and a truer picture of ability, so would prove useful for assessment and selection”. Relatedly, the study of Mann and van Ginneken (28) suggested that when presented with information regarding the relative age of players while simultaneously assessing performance by means of age-ordered shirt numbering, coaches and/or talent scouts were able to reduce the relative age bias in their assessments. As such, cueing differences in age might be especially appropriate for mitigating indirect effects, which typically emerge through social agents' behaviours.

Regarding feasibility, the majority of participants commented that “cueing differences in age” can be implemented “quickly and effectively” (Participant 2). Indeed, participants agreed that it is likely that it can be implemented immediately (% _agree_ = .92) without considering the macro sporting context (e.g., league structures and regulations) (7.8 ± 1.9; IQR = 1.5). However, given that these solutions mainly target the selection of players, which typically takes place on an annual basis, it might not address the direct advantages of being relatively older. For instance, Participant 2 also stated that: “It may not prevent certain players being disadvantaged during the game”, highlighting that the direct effects attributed to age-related differences are likely not addressed by this solution. This is also reflected in the relatively higher disagreement about cueing difference in age decreasing “the inflated expectations of player's ability from coaches and parents” (5.9 ± 2.3; IQR = 2.5). Therefore, it may be a more beneficial solution to use during momentary talent identification and selection activities (e.g., trials, talent identification events) rather than routine training and competition; or alternatively, in conjunction with other solutions to mitigate more direct effects. However, further research is required to substantiate these suggestions.

The most effective overall solution was “rotating cut-off dates”. This solution aims to balance RAEs by rotating the selection cut-off date (e.g., changing the cut-off date 3-months every year). This way, each player would spend some time as the oldest player amongst an age group, and some time as the youngest. Participants suggest that it does not address the age advantage *per se*, but does alter “player experiences of being the youngest and oldest on the team” (Participant 7). As such, participants generally agreed that this solution might decrease “the likelihood that relatively older players created false self-beliefs” (6.7 ± 1.7; IQR = 1.5) and “stakeholders over-estimating the playing ability of relatively older players” (6.3 ± 1.6; IQR = 1). As stated by Participant 5, this solution could potentially increase relatively younger players self-image, “as the peer group being used for comparisons would always be changing”. Although such statements remain hypothetical as rotating cut-off dates have not been researched of widely implemented in soccer, lessons could be learnt from the “birthday-banding” approach (i.e., athletes competing with and against those of the same age and move up to their next birthdate group on their birthday) used in the England Squash Talent Pathway, which has been attributed to the encouraging absence of RAEs across their cohorts (42). Importantly, however, while participants indicated that “the idea that players experience being the oldest and youngest at some point is one worth considering further” (Participant 4), they were hesitant and in disagreement regarding its feasibility (M = 4.6 ± 2,5, *K_alpha_* = .15). In particular, participants were doubtful about the logistics that “would have to be well thought out and communicated and understood very clearly” (Participant 2), as well as requiring buy-in from stakeholders (e.g., coaches, administrators, parents).

Although “submitting entry exemption” was ranked equal third with the same overall score as “rotating cut-off dates”, there was generally less agreement amongst the panellists regarding its utility (*K_alpha_* = .17 vs. *K_alpha_* = .12). Entry exemption enables players to “play-down”, which means that a player competes in competitions designed for younger age groups (43). Typically, eligibility criteria (e.g., youth league policies) do not allow players to play down, resulting in relatively younger players born closer to the cut-off date fixed to play with and against relatively older players. This solution, however, proposes easing the eligibility criteria, particularly for relatively younger players (e.g., players born in the second half of the selection year). As such, these children could potentially avoid the associated disadvantages of being younger, whilst also providing a more challenging environment for the older birth quartiles in the younger age group. Although yet to be empirically evaluated, anecdotal evidence has showed how some England international players played down in academy soccer during their development (44). Some participants, however, issued possible warnings regarding the stigma surrounding playing-down. For instance, Participant 1 suggested: “We would have to change the cultural interpretation associated with playing-up or playing-down. Playing-up is seen as good, playing-down as bad”.

Another solution that was ranked high on its effectiveness to mitigate direct RAEs was “categorising on characteristics other than age”. In particular, the participants considered grouping players on alternative criteria as a viable solution to address “feelings of incompetence while playing football for relatively younger players” (6.7 ± 2.0; IQR = 1.0), although disagreed more on the solution mitigating “the reliance of relatively older players on their maturational advantage” (6.7 ± 2.2; IQR = 2.0). Given the hypothesised developmental advantages that influence RAEs and the fact that relatively older players have had more time to grow and physically develop [e.g., (45–47)], it is expected that controlling for anthropometric and/or physical characteristics could mitigate RAEs. As such, alternative grouping criteria that have been suggested are height/weight categories or initiatives such as “bio-banding”, whereby players are grouped according to their level of maturation, often using their percentage of predicted adult height (48).

Closely related to using height and weight categories as a grouping strategy was “grouping based on chronological and biological age”. This solution suggests using the height of players relative to their peers as a criterion for grouping. Although, on average, this solution was rated more feasible (5.2 ± 2.5) and panel members were more in agreement (*K_alpha_* = .27) compared to “categorising on characteristics other than age” (4.4 ± 2.3), some participants were cautious to try and solve temporary, maturational advantages together with relative age differences. As one participant stated: “Maturity and RAE share about 8 percent variance in academy football” (Participant 1). In addition, for both “grouping based on chronological and biological age” (IQR = 1.5) and “categorising on characteristics other than age” (IQR = 1.25; %_agree_ = .92), participants perceived the buy-in from stakeholders (e.g., coaches, administrators, parents) working in youth soccer necessary. This was also the case for “delaying selection and deselection” (IQR = 2.0; %_agree_ = .83) and “testing objective skills” (IQR = 2.0; %_agree_ = .83).

## Discussion

The aim of this present study was to evaluate the potential direct and indirect effectiveness and feasibility of 13 proposed solutions to mitigate RAEs in youth soccer that were gathered in Part One of this project. Based on our modified e-Delphi approach, “cueing differences in age” was considered the most viable solution across both rounds. “Grouping based on chronological and biological age” and “rotating cut-off dates” were also perceived highly to mitigate direct and indirect RAEs as well as the most feasible to implement into youth soccer. Interestingly, these three solutions are from three different higher-order categories according to the taxonomy of Mann (26), which perhaps underscores the importance of designing hybrid approaches that target multiple aspects of RAEs.

Overall, “shifting cut-off dates” was rated the lowest solution, with “using corrective adjustments” and “testing objective skills” also rated lower than other approaches. With regards to effectiveness, “raising awareness of RAEs” was rated low but rated high on feasibility, whilst “cueing differences in age” and “modifying age bands” were rated differently on their effectiveness for direct and indirect RAEs, respectively. With regards to feasibility, on average, overall ratings were lower compared with effectiveness, highlighting some of the practical barriers that are associated with implementing relative age solutions. As an example, “rotating cut-off dates” was deemed effective in terms of mitigating RAEs, but rated lower on its feasibility. Taken together, our study suggests that potentially effective solutions to mitigate RAEs were generally considered less feasible to implement, whereas those that are possibly more feasible were generally considered less effective. This may highlight that a range of different approaches may be required to combat RAEs in soccer.

Results perhaps also suggest that the current organisational structures in youth soccer and its related activities (i.e., talent identification, selection, training, and competition) are a contributing factor to the ongoing presence RAEs. As such, any attempt to mitigate RAEs in youth soccer should also, at least to some extent, address the manner in which common practices in youth soccer are executed, whether through altering observer behaviour or adjusting competition structures. This might explain the lack of solutions that have been empirically tested or implemented. Moreover, this could potentially provide a rationale for the common disagreement between experts and the proposed solutions (i.e., only five out of 13 solutions reached consensus on more than one aspect regarding the effectiveness and feasibility of the respective solution).

To our knowledge, “cueing differences in age” is one of only two proposed solutions that have been empirically assessed to date in youth soccer (28). Through age-ordered shirt numbering, Mann and van Ginneken (28) showed that during a soccer selection task, scouts were able to control for the relative age of players when assessing their potential. In the study, players competed in an 8 vs. 8 match and wore shirt numbers that corresponded to their relative age (i.e., oldest player wearing number “1”, and the youngest wearing number “8”). Results indicated that when scouts were aware of the age-ordered shirt numbering, it successfully reduced the relative age bias of their player potential rankings. Interestingly, this approach has also been previously shown to moderate maturation biases between soccer players when scouts are assessing their potential (49). This suggests that when individuals are explicitly provided with important information on a player, it can positively support their decision making.

Explicitly cuing relative age could also go beyond age ordered bibs, such as listing player registers in chronological age order and clearly providing relative ages on player observation reports, although further research is required to test how effective this could be. While the methodology of Mann and van Ginneken (28) has, so far, only been applied with scouts having to rank youth players, the solution might also address other direct or indirect RAEs. Regularly playing with age-ordered shirt numbers could make players, parents, coaches, and other stakeholders aware that differences in ability might result from differences in age (26). As such, this could mitigate the inflated expectations from coaches and parents, or the false self-beliefs amongst relatively older teammates and peers, although these expectations remain mostly hypothetical to date and require further research.

To directly address the possible growth advantages of relatively older players (50, 51), results from our study suggest that grouping on alternative characteristics besides chronological age might be effective. However, given the plethora of indicators to group players (e.g., height and weight, cognitive and social maturity) and the accompanying burden of objectively assessing these indicators, this solution was rated low in terms of feasibility. A closely related solution that was rated higher for its feasibility, however, was “grouping based on chronological and biological age”. This solution proposes the use the “developmental birth dates” to group players. Developmental birth dates are estimated by comparing a player's stature with the normative growth curves for the player's population (e.g., Dutch boys aged 1–21 years). In a preliminary study by Helsen et al. (29), this method was applied to reallocate a group of Belgium youth soccer players. In their study, the traditional chronological age-grouping resulted in significant RAEs amongst these players, with most players born in the first quarter of the selection year. Importantly, however, following the reallocation of players based upon their developmental birth dates, the overrepresentation of players in birth quartile one (i.e., those born in the first three months of the annual selection year) disappeared, with player's birthdates almost evenly distributed across each birth quartile (∼25%). While this study shows promising results in terms of removing RAEs when grouping players, it is yet to be tested within competitions, and as a maximum age difference could exceed more than three years, it could prove difficult to implement and gain stakeholder buy-in.

It is also important to acknowledge that relative age and biological age are two independent constructs that can impact individuals differently (52). More specifically, relative age remains fixed whereas biological age can differ up to five years between those within the same chronological age group (53). In fact, a recent commentary warned that, while relative age and maturity differences are two important biases that play a role in talent development, they should be considered two separate processes (54). Each occurring at different timepoints in a player's developmental career, operating independent from another, and impacting individuals differently, thus two distinct solutions may be important to consider when implementing solutions to mitigate both RAEs and maturation biases (e.g., RAEs occur from entry into soccer at childhood, whereas biological age differences in boys occur during adolescence) (55). It is important to remember, though, that research on the interconnectedness between relative age and biological age is still limited, while exploration of possible independent and combined solutions of these phenomena is still in early stages.

Both “cueing differences in age” and “grouping based on chronological and biological age” can be readily implemented at an individual club level, and do not necessarily require adjusting competition structures. In addition, both solutions can coexist due to their different methods, which creates the possibility for a hybrid approach, whereby multiple solutions are implemented to mitigate RAEs (19). Both these solutions target a different approach to mitigate RAEs and are considered to vary in their effectiveness to alleviate direct and indirect effects (26). “Cueing differences in age” is expected to primarily mitigate indirect effects by clarifying relative age differences for individuals to adjust their assessment of players. In contrast, “grouping on chronological and biological age” introduces constraints beyond traditional birthyear grouping and, as such, attempts to mitigate the developmental advantages of relatively older players (i.e., direct effects). Despite the relative autonomy that comes with implementing both solutions, it has not yet been widely adopted. Although there are anecdotal cases of sports teams using age-ordered shirt numbering during their selection procedures (26), it will be important to evaluate these attempts to build on the current evidence.

Our results highlight that while several solutions to mitigate RAEs have been put forward, many of these were perceived difficult to implement. This might explain the relative lack of empirical work that has tested these solutions in real-world youth soccer settings. For instance, despite being the highest rated solution on effectiveness to mitigate both direct and indirect RAEs, “rotating cut-off dates” was only ranked tenth in terms of feasibility. One of the main reasons for this is the associated complexity for coaches and other stakeholders that occurs when regularly changing the cut-off date to group players into teams. Nevertheless, the Football Flanders [Voetbal Vlaanderen] (56) has recently announced that, from July 2025, they will introduce “rotating cut-off dates” within the calendar-year, by alternating the cut-off dates between January 1st to July 1st every six months. While the barrier to implementing this approach might be solved with clear communication and administration, other solutions carry potential unforeseen risks for athlete development. For example, solutions such as “submitting entry exemption” or “capping the average team age” might increase injury risk or break-up age group friendship groups (57, 58). Therefore, it is important to consider the unintended consequences that could come with widely implementing relative age solutions without evaluating their effectiveness and feasibility beforehand.

Although it is beyond the capacity of this discussion to critically review all 13 relative age solutions [see Part One for an overview of each proposed solution; (23)], there is some additional feedback from the experts to consider. With regards to the lowest rated solution, whilst “shifting cut-off dates” in different sports would make it possible for all children to experience a favourable cut-off date, it might not solve RAEs in soccer *per se*. In 1997, for example, the Belgian Soccer Federation changed the start of their cut-off date from August 1st to January 1st. This shift prompted an investigation from Helsen et al. (59) who explored the changes in the birthdate distributions throughout youth competitions for 1996–1997 compared to 1997–1998. Their findings revealed a shift of RAEs corresponded with the new cut-off dates, thus “shifting cut-off dates” will likely just shift the relative age distribution. Finally, “raising awareness of RAEs” was suggested as a highly feasible solution that requires minimal resources. However, considering the effectiveness, this solution was rated relatively low overall. Indeed, Helsen et al. (60) demonstrated that, over a period of 10 years, the magnitude of RAEs did not decrease in European soccer. As such, the authors concluded that, assuming education and attention regarding RAEs in soccer had taken place in that period, raising awareness may not be effective in mitigating RAEs.

Related to the relative lack of solutions that have been applied in practice is the concept of knowledge mobilisation (KMb). This concept refers to the process of taking research beyond the academic domain to have an impact in real-world settings (61). While many models and frameworks have been developed to describe the process of KMb, most strategies to translate knowledge from research to practice use three approaches (62). The first strategy entails “connecting” knowledge stakeholders, such as researchers and practitioners via “knowledge brokers” (63). These could be, for instance, sport's governing bodies that mobilise knowledge through coach education. For example, following interviews with seven talent identification experts, Andronikos et al. (64) showed how “raising awareness of RAEs” for the likes of coaches, scouts, and clubs, was perceived to be part of the controllable features available to eradicate them. The second strategy entails “disseminating” knowledge via (online) resources and easily accessible documentation. As an example, as part of this project, we have already shared our results via the KNVB website in an attempt to widely disseminate key findings (65). Lastly, a strategy to translate knowledge would be to facilitate “interactions”, such as participatory research. Specifically, actively involving all stakeholders (e.g., researchers, coaches, recruiters, policy makers, players, parents) seems a particularly fruitful approach to design, implement, and evaluate potential relative age solutions (19), which could prove a useful next step for the Royal Dutch Football Association (KNVB) Relative Age Solutions Project. Overall, researchers and practitioners should consider a KMb approach when seeking to moderate RAEs in youth soccer.

### Limitations and future directions

One of the limitations of this study is related to the e-Delphi design. Typically, the e-Delphi method consists of several iterative rounds with controlled feedback to achieve consensus. However, given the diverse group of panelists and the risk of participant attrition often occurring with Delphi studies (66), we aimed to minimise participant burden by priori setting the number of e-Delphi rounds to two. In addition, we reduced the duration of the study by enabling participants to immediately commence the second e-Delphi round, whereas panelists typically receive feedback from the previous rounds, which might alter their perspective. It is also important to understand the contextual variances of different youth soccer environments across the world. This may have resulted in variations in effectiveness and feasibility depending on the likes of resources available, knowledge uptake, and sport popularity. Therefore, those working in soccer are encouraged to recognise that there is no “copy and paste” template when it comes to solving RAEs, and that they should seek to comprehend potential solutions based on the contextual complexities of cultures, communities, and individual circumstances (67). However, since RAEs are pervasive in youth soccer across the world, attempts to minimise or remove them could be considered universal, with a range of different approaches proving useful to test and evaluate in different contexts.

Regarding future directions, as a first step, researchers, coaches, and policymakers can use our findings as a list of options that might moderate RAEs. As such, the outcomes of our study can serve as a starting point for governing bodies and soccer academies to test different options, whilst also providing considerations of the potential effects when implementing these solutions. For instance, the panellists highlighted the requirement of buy-in from different stakeholders for several solutions to be successful. We would, therefore, recommend that before adjusting competition structures, stakeholders should be informed about the potential changes and challenges. In addition, while the experts reached consensus on some of the items regarding the effectiveness and feasibility of potential RAEs, there was regular disagreement between them. This highlights the need for more empirical research to validate expert opinions. As such, we recommend creating real-world experiments to test the utility of these solutions. While such experiments are complex and require significant resources, the results of our study can be used to prioritise certain solutions. Relatedly, we recommend testing these solutions in a range of youth soccer settings, with researchers and universities working collaboratively with soccer organisations and industry stakeholders to support improved KMb (68).

## Conclusion

There are many empirical studies on RAEs in soccer that highlight its existence and emphasise the need mitigate such effects. However, only a few researchers have attempted to analyse ways in which RAEs can truly be moderated or removed. In light of the expert consensus and feedback regarding the effectiveness and feasibility of the 13 proposed relative age solutions presented in this study, an important next step will be to design, implement, and evaluate the highly rated solutions in practice. This will help capture the most effective and feasible approaches based upon the needs and context of different youth soccer environments.

## Data Availability

The raw data supporting the conclusions of this article will be made available by the authors, without undue reservation.

## References

[1] Grondin S Deshaies P Nault LP. Trimestres de naissance et participation au hockey et au volleyball. La Revue Québéecoise de L’Activité Physique. (1984) 2:97–103.

[2] Barnsley RH Thompson AH Barnsley PE. Hockey success and birthdate: the RAE. Can Assoc Health Phys Educ Recreat. (1985) 51:23–8.

[3] Cobley S Baker J Wattie N McKenna J. Annual age-grouping and athlete development. Sports Med. (2009) 39(3):235–56. 10.2165/00007256-200939030-00005 19290678

[4] Musch J Grondin S. Unequal competition as an impediment to personal development: a review of the relative age effect in sport. Dev Rev. (2001) 21(2):147–67. 10.1006/drev.2000.0516

[5] Smith KL Weir PL Till K Romann M Cobley S. Relative age effects across and within female sport contexts: a systematic review and meta-analysis. Sports Med. (2018) 48(6):1451–78. 10.1007/s40279-018-0890-8 29536262

[6] Dixon J Horton S Chittle L Baker J. Relative Age Effects in Sport: International Perspectives (1st ed.). New York, NY: Routledge (2020). 10.4324/9781003030737

[7] Kelly A Côté J Jeffreys M Turnnidge J. Birth Advantages and Relative Age Effects in Sport: Exploring Organizational Structures and Creating Appropriate Settings (1st ed.). New York, NY: Routledge (2021). 10.4324/9781003163572

[8] Breznik K Law KMY. Relative age effect in mind games: the evidence from elite chess. Percept Mot Skills. (2016) 122(2):583–94. 10.1177/0031512516640957 27166336

[9] Helsen WF Baker J Schorer J Steingröver C Wattie N Starkes JL. Relative age effects in a cognitive task: a case study of youth chess. High Abil Stud. (2016) 27(2):211–21. 10.1080/13598139.2016.1242063

[10] Delorme N Raspaud M. The relative age effect in young French basketball players: a study on the whole population. Scand J Med Sci Sports. (2009) 19(2):235–42. 10.1111/j.1600-0838.2008.00781.x 18298612

[11] Kelly AL Sáiz SLJ Calvo AL De La Rubia A Jackson DT Jeffreys MA Relative age effects in basketball: exploring the selection into and successful transition out of a national talent pathway. Sports. (2021) 9(7):101. 10.3390/sports9070101 34357935 PMC8309713

[12] Kelly AL Brown T Reed R Côté J Turnnidge J. Relative age effects in male cricket: a personal assets approach to explain immediate, short-term, and long-term developmental outcomes. Sports. (2022) 10(3):39. 10.3390/sports10030039 35324648 PMC8949933

[13] Radnor JM Oliver JL Dobbs I Wong M Brown TW Lloyd RS Selection into youth cricket academies: the influence of relative age and maturity status. J Sports Sci. (2023) 41(3):272–9. 10.1080/02640414.2023.2208924 37163466

[14] Romann M Rössler R Javet M Faude O. Relative age effects in Swiss talent development—a nationwide analysis of all sports. J Sports Sci. (2018) 36(17):2025–31. 10.1080/02640414.2018.1432964 29392997

[15] Brustio PR Lupo C Ungureanu AN Frati R Rainoldi A Boccia G. The relative age effect is larger in Italian soccer top-level youth categories and smaller in serie A. PLoS One. (2018) 13(4):e0196253. 10.1371/journal.pone.0196253 29672644 PMC5909613

[16] Helsen WF Van Winckel J Williams AM. The relative age effect in youth soccer across Europe. J Sports Sci. (2005) 23(6):629–36. 10.1080/02640410400021310 16195011

[17] Kelly AL Williams CA. Physical characteristics and the talent identification and development processes in youth soccer: a narrative review. Strength Cond J. (2020) 42(6):15–34. 10.1519/SSC.0000000000000576

[18] Yagüe JM de la Rubia A Sánchez-Molina J Maroto-Izquierdo S Molinero O. The relative age effect in the 10 best leagues of male professional football of the union of European football associations (UEFA). J Sports Sci Med. (2018) 17(3):409–16. 30116114 PMC6090398

[19] Webdale K Baker J Schorer J Wattie N. Solving sport’s ‘relative age’ problem: a systematic review of proposed solutions. Int Rev Sport Exerc Psychol. (2019) 13(1):187–204. 10.1080/1750984x.2019.1675083

[20] Smith KL Weir PL. An examination of relative age and athlete dropout in female developmental soccer. Sports. (2022) 10(5):79. 10.3390/sports10050079 35622488 PMC9148022

[21] Hancock DJ Adler AL Côté J. A proposed theoretical model to explain relative age effects in sport. Eur J Sport Sci. (2013) 13(6):630–7. 10.1080/17461391.2013.775352 24251740

[22] Wattie N Schorer J Baker J. The relative age effect in sport: a developmental systems model. Sports Med. (2015) 45(1):83–94. 10.1007/s40279-014-0248-9 25169442

[23] Kelly AL Zwenk F Mann D Verbeek V. The royal Netherlands football association (KNVB) relative age solutions project—part one: a call to action. Front Sports Act Living. (2025) 7:1546829. 10.3389/fspor.2025.1546829 40276309 PMC12018424

[24] Ford PR Bordonau JLD Bonanno D Tavares J Groenendijk C Fink C A survey of talent identification and development processes in the youth academies of professional soccer clubs from around the world. J Sports Sci. (2020) 38(11-12):1269–78. 10.1080/02640414.2020.1752440 32378447

[25] Jackson RC Comber G. Hill on a mountaintop: a longitudinal and cross-sectional analysis of the relative age effect in competitive youth football. J Sports Sci. (2020) 38:1352–8. 10.1080/02640414.2019.1706830 31916503

[26] Mann D. Approaches to help coaches and talent scouts overcome relative age effects. In: Dixon JC Horton S Chittle L Baker J, editors. Relative Age Effects in Sport: International Perspectives. New York, NY: Routledge (2020). p. 117–35.

[27] Kelly AL Turnnidge J. Group banding strategies in children’s organised sport: looking beyond fixed chronological age. In: Toms MR Jeanes R, editors. Handbook of Coaching Children in Sport. New York, NY: Routledge (2023). p. 303–14.

[28] Mann DL van Ginneken PJMA. Age-ordered shirt numbering reduces the selection bias associated with the relative age effect. J Sports Sci. (2017) 35(8):784–90. 10.1080/02640414.2016.1189588 27238077

[29] Helsen WF Thomis M Starkes JL Vrijens S Ooms G MacMaster C Leveling the playing field: a new proposed method to address relative age- and maturity-related bias in soccer. Front Sports Act Living. (2021) 3:635379. 10.3389/fspor.2021.635379 33748755 PMC7969981

[30] Linstone HA Turoff M. The Delphi Method. Reading, MA: Addison-Wesley (1975).

[31] Beiderbeck D Frevel N Von Der Gracht HA Schmidt SL Schweitzer VM. Preparing, conducting, and analyzing Delphi surveys: cross-disciplinary practices, new directions, and advancements. MethodsX. (2021) 8:101401. 10.1016/j.mex.2021.101401 34430297 PMC8374446

[32] Manyara AM Purvis A Ciani O Collins GS Taylor RS. Sample size in multistakeholder Delphi surveys: at what minimum sample size do replicability of results stabilize? J Clin Epidemiol. (2024) 174:111485. 10.1016/j.jclinepi.2024.111485 39069013 PMC7617918

[33] Hsu C Sandford BA. The Delphi technique: making sense of consensus. Pract Assess Res Eval. (2007) 12(10):10. 10.7275/pdz9-th90

[34] Nasa P Jain R Juneja D. Delphi methodology in healthcare research: how to decide its appropriateness. World J Methodol. (2021) 11(4):116–29. 10.5662/wjm.v11.i4.116 34322364 PMC8299905

[35] Donohoe HM Needham RD. Moving best practice forward: Delphi characteristics, advantages, potential problems, and solutions. Int J Tour Res. (2009) 11(5):415–37. 10.1002/jtr.709

[36] De Meyer D Kottner J Beele H Schmitt J Lange T Van Hecke A Delphi procedure in core outcome set development: rating scale and consensus criteria determined outcome selection. J Clin Epidemiol. (2019) 111:23–31. 10.1016/j.jclinepi.2019.03.011 30922885

[37] Diamond IR Grant RC Feldman BM Pencharz PB Ling SC Moore AM Defining consensus: a systematic review recommends methodologic criteria for reporting of Delphi studies. J Clin Epidemiol. (2014) 67(4):401–9. 10.1016/j.jclinepi.2013.12.002 24581294

[38] R Core Team. R: A Language and Environment for Statistical Computing (4.4.0). Vienna: R Foundation for Statistical Computing (2024). Available at: http://www.r-project.org/ (Accessed November 12, 2024).

[39] Gamer M. irr: various coefficients of interrater reliability and agreement. R package version 0.84.1 (2005). Available at: https://cran.r-project.org/web/packages/irr (Accessed March 4, 2025).

[40] Hayes AF Krippendorff K. Answering the call for a standard reliability measure for coding data. Commun Methods Meas. (2007) 1(1):77–89. 10.1080/19312450709336664

[41] Okoli C Pawlowski SD. The Delphi method as a research tool: an example, design considerations and applications. Inf Manag. (2004) 42(1):15–29. 10.1016/j.im.2003.11.002

[42] Kelly AL Jackson DT Taylor JJ Jeffreys MA Turnnidge J. Birthday-banding” as a strategy to moderate the relative age effect: a case study into the England squash talent pathway. Front Sports Act Living. (2020) 2:573890. 10.3389/fspor.2020.573890 33345136 PMC7739587

[43] Kelly AL Wilson MR Gough LA Knapman H Morgan P Cole M A longitudinal investigation into the relative age effect in an English professional football club: exploring the ‘underdog hypothesis’. Sci Med Football. (2020b) 4(2):111–8. 10.1080/24733938.2019.1694169

[44] Kelly AL Goldman DE Côté J Turnnidge J. Playing-up and playing-down: conceptualising a ‘flexible chronological approach’. In: Kelly AL, editor. Talent Identification and Development in Youth Soccer: A Guide for Researchers and Practitioners. New York, NY: Routledge (2023). p. 152–66.

[45] Drenowatz C Ferrari G Greier K Hinterkörner F. Relative age effect in physical fitness during the elementary school years. Pediatr Rep. (2021) 13(2):322–33. 10.3390/pediatric13020040 34201263 PMC8293459

[46] Roberts SJ Boddy LM Fairclough SJ Stratton G. The influence of relative age effects on the cardiorespiratory fitness levels of children age 9 to 10 and 11 to 12 years of age. Pediatr Exerc Sci. (2012) 24(1):72–83. 10.1123/pes.24.1.72 22433266

[47] Wattie N Tietjens M Schorer J Ghanbari M-C Strauss B Seidel I Does relative age influence motor test performance of fourth grade pupils? Eur Phy Educ Rev. (2014) 20(3):398–406. 10.1177/1356336X14534363

[48] Malina RM Cumming SP Rogol AD Coelho-E-Silva MJ Figueiredo AJ Konarski JM Bio-banding in youth sports: background, concept, and application. Sports Med. (2019) 49(11):1671–85. 10.1007/s40279-019-01166-x 31429034

[49] Lüdin D Donath L Cobley S Mann D Romann M. Player-labelling as a solution to overcome maturation selection biases in youth football. J Sports Sci. (2022) 40(14):1641–7. 10.1080/02640414.2022.2099077 35969578

[50] Gil SM Badiola A Bidaurrazaga-Letona I Zabala-Lili J Gravina L Santos-Concejero J Relationship between the relative age effect and anthropometry, maturity and performance in young soccer players. J Sports Sci. (2014) 32(5):479–86. 10.1080/02640414.2013.832355 24050650

[51] Sæther SA Peterson T Matin V. The relative age effect, height and weight characteristics among lower and upper secondary school athletes in Norway and Sweden. Sports. (2017) 5(4):92. 10.3390/sports5040092 29910452 PMC5969021

[52] Radnor JM Staines J Bevan J Cumming SP Kelly AL Lloyd RS Maturity has a greater association than relative age with physical performance in English male academy soccer players. Sports. (2021) 9(12):171. 10.3390/sports9120171 34941809 PMC8705996

[53] Hill M Scott S Malina RM McGee D Cumming SP. Relative age and maturation selection biases in academy football. J Sports Sci. (2020) 38(11-12):1359–67. 10.1080/02640414.2019.1649524 31366286

[54] Towlson C MacMaster C Parr J Cumming S. One of these things is not like the other: time to differentiate between relative age and biological maturity selection biases in soccer? Sci Med Football. (2021) 6(3):273–6. 10.1080/24733938.2021.1946133 35866421

[55] Johnson A Farooq A Whiteley R. Skeletal maturation status is more strongly associated with academy selection than birth quarter. Sci Med Football. (2017) 1(2):157–63. 10.1080/24733938.2017.1283434

[56] Football Flanders. 11 novelties youth soccer (2024). Available at: https://vvsite-prod.rbfa.be/club/jeugdsport-18/11-nieuwigheden-jeugdvoetbal (Accessed May 8, 2024).

[57] Campbell EC Bracewell PJ Blackie E Patel AK. The impact of auckland junior rugby weight limits on player retention. J Sport Health Res. (2018) 10(2):317–26.

[58] Moir J. Size does matter, 71 kg 10-year-old gets told (2013). Available at: http://www.stuff.co.nz/sport/rugby/8476408/Siz%20e-does-matter-71kg-10-year-old-gets-told (Accessed October 18, 2024).

[59] Helsen WF Starkes JL Van Winckel J. Effect of a change in selection year on success in male soccer players. Am J Hum Biol. (2000) 12(6):729–35. 10.1002/1520-6300(200011/12)12:6<729::AID-AJHB2>3.0.CO;2-7 11534065

[60] Helsen WF Baker J Michiels S Schorer J Van Winckel J Williams AM. The relative age effect in European professional soccer: did ten years of research make any difference? J Sports Sci. (2012) 30(15):1665–71. 10.1080/02640414.2012.721929 23005576

[61] Bayley JE Phipps D Batac M Stevens E. Development of a framework for knowledge mobilisation and impact competencies. Evid Policy. (2017) 14(4):725–38. 10.1332/174426417X14945838375124

[62] Ward V. Why, whose, what and how? A framework for knowledge mobilisers. Evid Policy. (2016) 13(3):477–97. 10.1332/174426416X14634763278725

[63] Jacobzone S Picalarga S Public O Directorate G. Mobilising Evidence to Enhance the Effectiveness of Child Well-Being Policies. OECD Working Papers on Public Governance (2023). 10.1787/faeb9a0d-en.

[64] Andronikos G Elumaro AI Westbury T Martindale RJ. Relative age effect: implications for effective practice. J Sports Sci. (2016) 34(12):1124–31. 10.1080/02640414.2015.1093647 26417709

[65] KNVB. Results of research into solutions for the birth month effect (2023). Available at: https://www.knvb.nl/nieuws/assist-trainers/assist-trainers/67542/uitkomsten-onderzoek-naar-oplossingen-geboortemaandeffect (Accessed April 10, 2023).

[66] Hall DA Smith H Heffernan E Fackrell K. Recruiting and retaining participants in e-Delphi surveys for core outcome set development: evaluating the COMiT’ID study. PLoS One. (2018) 13(7):e0201378. 10.1371/journal.pone.0201378 30059560 PMC6066228

[67] O’Sullivan M Vaughan J Woods CT Davids K. There is no copy and paste, but there is resonation and inhabitation: integrating a contemporary player development framework in football from a complexity sciences perspective. J Sports Sci. (2023) 43(1):99–108. 10.1080/02640414.2023.2288979 38095157

[68] Kelly AL Turnnidge J. From knowledge to action: bridging the gap between research and practice in youth soccer. In: Kelly AL, editor. Talent Identification and Development in Youth Soccer: A Guide for Researchers and Practitioners. New York, NY: Routledge (2023). p. 349.

