# Supplementary material for: The Royal Netherlands Football Association (KNVB) relative age solutions project—part two: an adapted e-Delphi study
Source: Front Sports Act Living. 2025 May 29;7:1565819. doi: 10.3389/fspor.2025.1565819 (PMC12159537; doi:10.3389/fspor.2025.1565819)
Supplement: Supplementary file 1 [file Datasheet1.pdf]

A DELPHI STUDY TO EVALUATE SOLUTIONS PROPOSED FOR RELATIVE AGE EFFECTS IN DUTCH YOUTH FOOTBALL

DELPHI ROUND 1

Round 1: The ability of each solution to mitigate direct and indirect RAEs

In round one, the participants are asked to evaluate the likelihood that each solution would be successful in mitigating the direct and indirect RAEs in football. These direct and indirect effects refer to the benefits experienced by the relatively older players (or disadvantages, in the case of relatively younger players). In addition, the participants are encouraged to provide a justification for their rating for each presumption. The following presumptions have been developed for participants to assess:

"Direct effects"

This solution …

1. … decreases the likelihood of relatively younger players performing worse than their peers during competition.

   Not very likely O O O O O O O O O **Very likely**

2. … decreases the likelihood of relatively older players experiencing greater levels of self-efficacy.

   Not very likely O O O O O O O O O **Very likely**

3. … decreases the likelihood of relatively younger players experiencing feelings of incompetence while playing football.

   Not very likely O O O O O O O O O **Very likely**

4. … decreases the likelihood of relatively older players relying on their maturational advantage (e.g. increased endurance, speed) instead of developing other skills (e.g. technical, tactical, and psychosocial) during training and matches.

Not very likely O O O O O O O O O **Very likely**

5. … decreases the likelihood of relatively older players creating false self-beliefs originating from their initial age advantage.

   Not very likely O O O O O O O O O **Very likely**

6. … decreases the likelihood of relatively older players having higher levels of involvement in games during training and competition.

   Not very likely O O O O O O O O O **Very likely**

"Indirect effects"

7. … decreases the likelihood of a selection bias caused by relatively older players being judged as more talented by coaches and/or talent scouts.

   Not very likely O O O O O O O O O **Very likely**

8. … decreases the likelihood of relatively older players being selected into better learning environment, such as representative or academy teams (i.e., above local-club/recreational level).

   Not very likely O O O O O O O O O **Very likely**

9. … decreases the likelihood that parents would delay the starting age that relatively younger players begin to play in organised youth football.

   Not very likely O O O O O O O O O **Very likely**

10. … decreases the likelihood that coaches and parents have inflated expectations for relatively older players from an over-estimation of their playing ability.

    Not very likely O O O O O O O O O **Very likely**

Participants are also encouraged to provide a justification for their rating for each attribute.

DELPHI ROUND 1

Round 2: Feasibility to apply and implement solutions in junior Dutch football leagues

A thorough understanding of practical feasibility of the proposed solutions would prove useful to eventually find the best intervention(s) to reduce RAEs in Dutch football. As such, in round two, the participants will be questioned on how feasible every proposed solution is to apply and implement in Dutch youth football leagues.

This solution …

1. … is applicable across all developmental levels (e.g., from recreational to representative levels) of youth football.

   Not very likely O O O O O O O O O **Very likely**

2. … requires significant financial resources (e.g., extra teams) at an individual club level

   Not very likely O O O O O O O O O **Very likely**

3. … requires significant physical resources (e.g., extra (parent-)coaches) at an individual club level.

   Not very likely O O O O O O O O O **Very likely**

4. … is only applicable at levels of youth football that are above local club-level.

   Not very likely O O O O O O O O O **Very likely**

5. … could be initiated by individual clubs or teams without considering the macro sporting context (e.g., league structures and regulations).

   Not very likely O O O O O O O O O **Very likely**

6. … does require buy-in from stakeholders (e.g., coaches, administrators, parents) working in youth football for success.

   Not very likely O O O O O O O O O **Very likely**

7. … is expected to yield positive results on a short time-scale (i.e., within one season).

   Not very likely O O O O O O O O O **Very likely**

8. … does allow for ‘immediate’ implementation (i.e., within the next season) across specific or multiple age-categories.

Not very likely O O O O O O O O O **Very likely**

9. … could result in players changing teams during a competitive season.

Not very likely O O O O O O O O O **Very likely**

10. … enhances (implicitly or explicitly) the way that a coach will focus on a player’s long-term development.

Not very likely O O O O O O O O O **Very likely**
